# Integrated Use of Furnace Bottom Ash as Fine Aggregate and Cement Replacement for Sustainable Mortar Production

**DOI:** 10.3390/ma17153834

**Published:** 2024-08-02

**Authors:** Waiching Tang, Ali M. Onaizi, Sagheer A. Onaizi, Umer Sajjad, Yanju Liu

**Affiliations:** 1 School of Architecture and Built Environment, The University of Newcastle, University Drive, Callaghan, NSW 2308, Australia; ali.onaizi@uon.edu.au (A.M.O.); umer.sajjad@newcastle.edu.au (U.S.); 2 Department of Chemical Engineering, King Fahd University of Petroleum and Minerals, Dhahran 31261, Saudi Arabia; onaizi@kfupm.edu.sa; 3 Interdisciplinary Research Center for Hydrogen Technologies and Carbon Management, King Fahd University of Petroleum and Minerals, Dhahran 31216, Saudi Arabia; 4 Global Centre for Environmental Remediation, The University of Newcastle, University Drive, Callaghan, NSW 2308, Australia; yanju.liu@newcastle.edu.au

**Keywords:** furnace bottom ash, cement replacement, fine aggregate replacement, mechanical properties, environmental impact

## Abstract

Recycling fly ash (FA) and furnace bottom ash (FBA) help with reducing greenhouse gas emissions, conserving natural resources, and minimizing waste accumulation. However, research on recycling FBA is progressing more slowly compared to FA. This research aims to investigate the combined use of FBA as a replacement for both fine aggregate and cement and its influence on the performance of mortar. The findings indicated that incorporating 25% FBA as a fine aggregate replacement and 10% or 20% ground FBA (GFBA) as a cement replacement significantly enhanced compressive strength after 28 and 56 days. Flexural strength was comparable to control mortar at 28 days and superior at 56 days. However, increasing the FBA content beyond 25% as a fine aggregate replacement reduced workability and increased porosity, which negatively affected mechanical performance and water absorption. Microstructural analyses revealed denser and more compact structures in the mortar with combined FBA replacement for both fine aggregate and cement, specifically 25% as a fine aggregate replacement and 10% and 20% as cement replacements. Optimal performance was noted in mixtures with Ca/Si and Ca/Al ratios within the ranges of 1.8–1.5 and 0.24–0.19, respectively. Trace element leaching analysis has not shown significant differences between GFBA, FA, and OPC. Regarding environmental impact assessment, using FBA as a fine aggregate replacement did not show a significant reduction in CO_2_ emissions, but replacing cement with FBA reduced emissions remarkably. Generally, using FBA as a replacement for both fine aggregate and cement in mortar enhances compressive and flexural strengths at optimal levels, promotes sustainability by reducing landfill waste and CO_2_ emissions, and supports cleaner production practices despite some workability challenges.

## 1. Introduction

Due to the economic advantages of coal, many countries still rely on the use of coal as the main source of electrical power generation [1,2]. The global proven coal reserves exceeded 1074 billion tonnes in 2020, enough to last more than 130 years at present global production rates [3,4,5]. According to the Statistical Review of World Energy by BP 2022 [6], global coal production increased from 6087.1 Mt in 2005 to 8185.6 Mt in 2012, with an annual growth rate of around 262 Mt%. The peak production was 8256.1 Mt in 2013, but this then declined to 7476.0 Mt in 2016 due to climate change awareness. Production rose again from 2017, reaching 8172.6 Mt in 2021, driven by economic growth in developing countries like China, India, and ASEAN. Australia ranks third globally, after the US and Russia, with 150.227 billion tonnes, about 14% of global reserves [5]. Coal demand is expected to rise this decade, increasing Coal Combustion Residues (CCPs) [5,7]. In the case of Australia, since May 2022, the Federal Environment Minister has approved four new coal mines, and there are 25 additional proposals for new or expanded coal mines currently waiting for federal government approval [8]. That means that the demand on coal is expected to continue rising at least during this decade [9]. Every year, Australian coal-fired power stations produce 12 million tonnes of ash from burning coal [10]. Coal ash accounts for 18% of Australia’s entire solid waste generation, which represents 500 kg per capita annually [10]. According to the World of Coal Ash (WOCA), Asian nations generate over 66% of global coal waste, followed by Europe and the Americas [11,12]. China produces around 395 Mt of coal waste, the US 118 Mt, and India 105 Mt [12]. China and the US recycle about 80% of coal fly ash, while India recycles 78% [13]. Most recycled quantities are used in the building and construction sectors, though specific data on recycled FBA is lacking [12].

Traditional disposal methods, involving open land areas or water bodies, exacerbate environmental pollution through air, water, and soil contamination. Therefore, devising effective solutions to effectively manage these vast amounts of coal ash is essential for sustainable development in Australia. Similar to fly ash (FA), recycling furnace bottom ash (FBA) meets sustainability goals by decreasing greenhouse gas (GHG) emissions, conserving natural resources, and minimizing waste generation. However, it is noteworthy that research progress on FBA is lagging behind that of FA. Zhou et al. [14] identify three main reasons why researchers tend to prioritize FA over FBA: (1) FBA production is significantly lower than FA, comprising only about 20–30% of the total coal ash generated; (2) FBA exhibits less leaching of toxic elements compared to FA; and (3) the heterogeneous nature of FBA complicates its recycling. Nonetheless, appropriate treatments such as sieving and grinding can mitigate many of the quality issues currently hindering FBA recycling.

In the case of using FBA as a sand replacement, most previous studies have noted a decrease in the workability of cementitious composites, and the reduction in workability increases with higher levels of replacement [15,16,17,18,19]. When FBA is utilized as a cement substitute, previous studies have shown contradictory results. Some research [20,21] reported an increase in workability, while others [22,23,24,25] observed a decrease. It is crucial to highlight that the impact of FBA on workability is influenced by several factors, including the physical and morphological properties of FBA, the mix design, and the replacement ratios [7]. Additionally, the irregular shape of FBA increases friction, and its porosity leads to higher water absorption, further reducing the mixture’s workability [15,17,26]. Also, the workability of concrete and mortar containing FBA as a cement substitute is affected by multiple factors, such as the grinding method, fineness and gradation, type and source of the coal ash, and replacement ratios [7,20,21,25,27,28].

Similarly, previous studies have reported inconsistent findings regarding the compressive strength of mortar and concrete incorporating FBA as a fine aggregate replacement, especially in the early stages. For example, Hasim et al. [29] and Rafieizonooz et al. [30] found that concrete mixes with FBA ranging from 50 to 100% as fine aggregate replacements showed similar compressive strength to that of the control specimen after 28 curing days. In contrast, other studies [17,18,31] reported a reduction in compressive strength for all replacement levels (from 20 to 100%) and at all curing days. Several factors significantly influence strength development, including the replacement level, the type of sand being replaced, and the treatment applied to the FBA before use. Soman et al. [32], Nanda and Rou [19], and Rafieizonooz et al. [18] observed similar trends in flexural and splitting tensile strengths. They reported a slight initial reduction in both flexural and splitting tensile strengths when FBA was used as a fine aggregate replacement, but these strengths improved with extended curing time.

When using FBA as cement replacement, several previous studies [20,21,23,24,27,33,34,35,36,37,38,39] have presented inconsistent findings regarding the compressive strength of cementitious composites containing 5–40% FBA as a cement replacement at early stages. However, most of those studies demonstrated acceptable outcomes in later stages. The compressive strength fluctuation generally ranged from a reduction of 30% to an increase of up to 10% at 28 days compared with the control samples. It is worth mentioning that the majority of studies revealed that the compressive strength performance at replacement levels of 10 and 15% was mostly similar to that of the control samples at 28 days, whereas a reduction was evident at higher replacement levels (e.g., at 30%). A similar trend to that of compressive strength was observed for flexural and splitting tensile strengths [24,25,34]. Despite the above-mentioned studies, the utilization of FBA for fine aggregate and cement replacement is still controversial, requiring more in-depth and well-designed studies to resolve the contradictory observations. While previous studies have explored the use of FBA as either cementitious materials or fine aggregate separately, this study focuses on its dual use as both a fine aggregate and cement replacement—a relatively underexplored area. This dual replacement approach aims to enhance the sustainability of mortar. Additionally, the results of the available studies are still contradictory, necessitating further research and investigation. This research aims to determine the optimal replacement ratios and their effects on compressive and flexural strength, workability, porosity, and environmental impact, reinforcing insights into the feasibility and benefits of FBA recycling in cementitious composite production. The significance of this study aligns with several Sustainable Development Goals (SDGs), including SDGs 12 and 13 for responsible consumption and production and SDG 9 for sustainable industrialization and infrastructure innovation. By incorporating FBA in cleaner production applications, particularly in the construction materials industry, we address the challenges of handling and disposing of FBA. This promotes recycling practices, reduces health and social risks from landfill contamination, and contributes to a healthier environment while significantly reducing carbon emissions in the construction sector.

## 2. Materials and Methods

### 2.1. Materials Preparation

**Figure 1:**
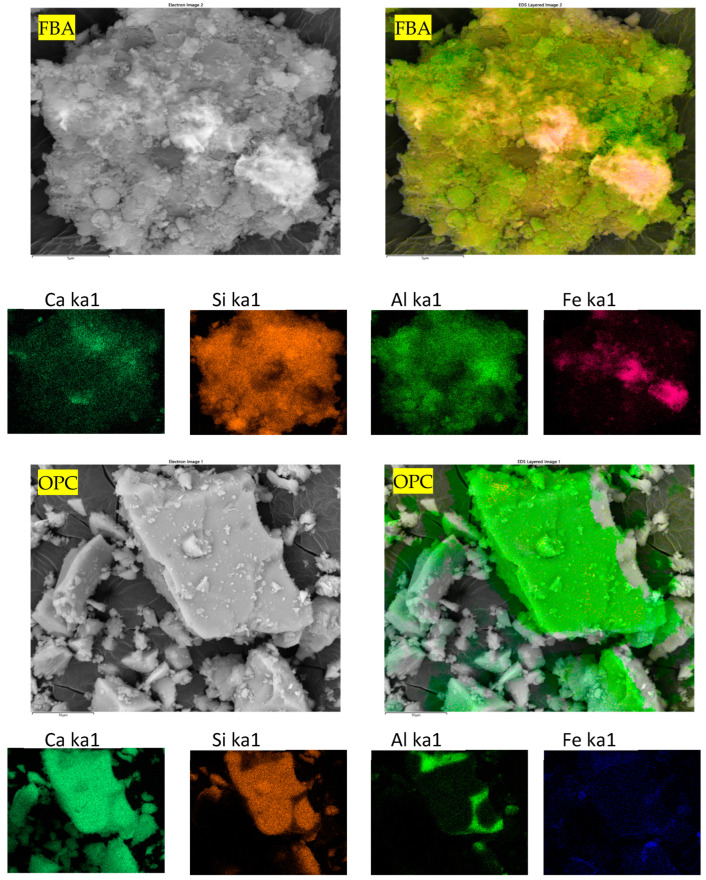
SEM and EDS images of GFBA and OPC.

**Figure 2:**
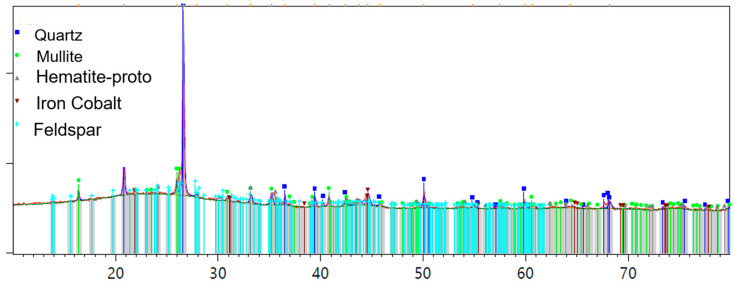
XRD patterns of ground FBA.

Traditional ordinary Portland cement (OPC) was obtained from Boral company, Sydney, Australia, conforming to AS 3972 [40] for Type GP and FBA from Vale Point Power Station in NSW were used as the raw materials. To prepare FBA for use as a cement substitute, the FBA was oven-dried for 24 h at 105 °C and sieved using a 2.36 mm sieve. Then, ball milling was conducted using an Across International PQ series planetary ball mill, with the ball milling mode set to unidirectional. A constant speed of 450 rpm was maintained for a grinding period of 30 min. The chemical compositions of OPC and ground FBA (GFBA) were then determined by XRF, as shown in Table 1. OPC primarily consists of CaO (70.91%), with Al2O_3_, SiO_2_, and Fe_2_O_3_ percentages of 4.64%, 15.30%, and 3.4%, respectively. In contrast, GFBA is mainly composed of SiO_2_, Al_2_O_3_, and Fe_2_O_3_, which collectively represent around 84.9% of its total mass. The findings from the XRF analysis were reinforced by the EDS analysis shown in Figure 1, which indicates SiO_2_, Fe_2_O_3_, and Al_2_O_3_ as the dominant oxides in GFBA. It is evident that GFBA complies with the ASTM C618 [41] requirements for Type F pozzolanic materials, especially fly ash (FA), as well as the AS/NZS 3582.1: 2016 [42] standards. The mean sum of its pozzolanic content (SiO_2_ + Al_2_O_3_ + Fe_2_O_3_) exceeds 70%, achieving 86.7% of the total weight ratio in its chemical composition. Additionally, its calcium content is 2.40%, which falls within the ASTM C618 and AS/NZS 3582.1: 2016 requirements for Type F. Specifically, ASTM C618 sets a maximum of 5% calcium content, while AS/NZS 3582.1: 2016 stipulates it should not exceed 10% (25% for New Zealand specifications) for the three grades (I, II, and Special grade). Figure 2 shows the XRD patterns of FBA, with a semi-quantitative XRD analysis conducted using the EMPYREAN diffractometer system (Cu Kα 1.54 Å) and PANalytical Data Collector software v.4.7. The analysis covered a 2θ range from 10.02° to 79.95°, with a step size of 0.0390° and scan step time of 195.585 s. HighScore Plus software v5.2, along with the ICDD database, was used for peak fitting and matching/searching, enabling semi-quantification of the sample’s mineralogical composition. The XRD analysis showed that GFBA primarily comprises silicon oxide (60%), mullite (27%), Feldspar (9%), and others constituents such as Hematite-Proto and Iron Cobalt.

**Figure 3:**
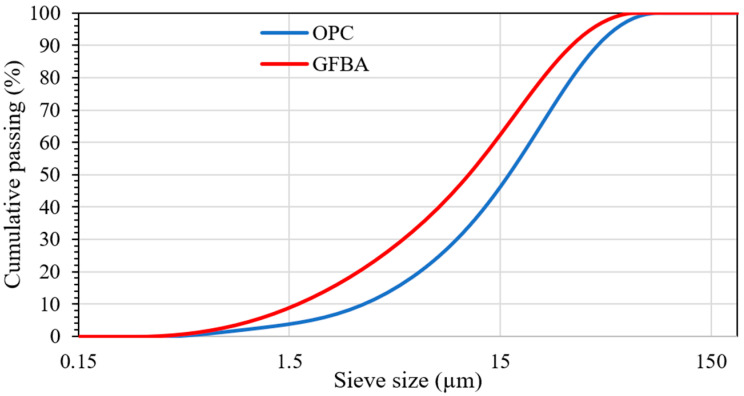
Particle size distribution of binder components.

Figure 3 illustrates the particle size distribution, while Table 2 presents the physical properties of OPC and GFBA. The properties of particle size distribution and specific surface areas were determined using MasterSizer2000 (Malvern Panalytical Ltd., Malvern, Worcestershire, UK) with water as the dispersant medium, as both materials dissolve easily in water. Additionally, a helium gas pycnometer was used to determine specific gravity. Three samples of each material were measured to determine specific gravity, particle size distribution, and specific surface area, and the averages were taken. The densities of OPC and GFBA were 3.12 and 2.45 g/cm^3^, respectively, while the specific surface areas were 8650 and 9857 cm^2^/g.

**Figure 4:**
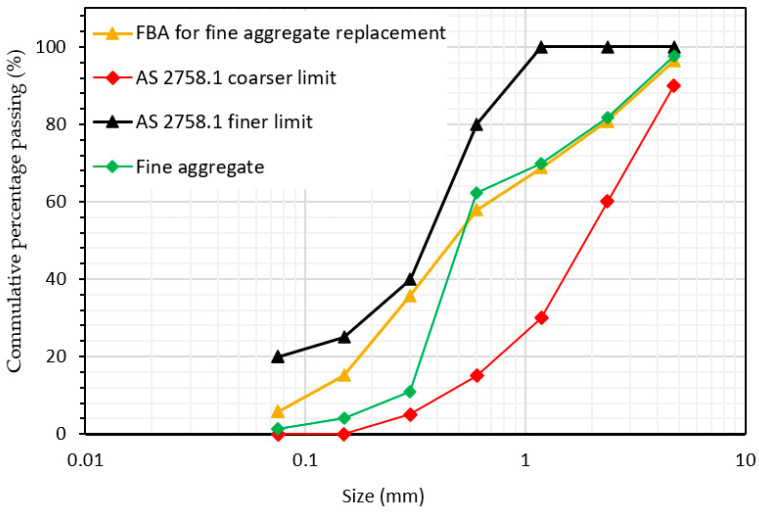
Particle size distribution of FBA and fine aggregates.

The fine aggregate consisted of a combination of river sand and fine crushed stones in a 1:1 ratio, sourced locally and sieved through a 4.75 mm sieve. For FBA utilized as a replacement for fine aggregate, it was oven-dried for 24 h at 105 °C and sieved through a 4.75 mm sieve. The particle size distribution of FBA and the fine aggregate, compared to the upper and lower limits of AS 2758.1 [43], is presented in Figure 4. The physical properties of FBA as a fine aggregate alternative and the fine aggregate are shown in Table 3. The fineness moduli of the combinations of fine aggregates and FBA were 4.7 and 4.4, respectively. The specific gravity and water absorption of FBA and the fine aggregate were also determined in accordance with AS 1141.5 [44], while the densities and moisture content were measured according to AS 1141.4 [45] and AS 3583.2 [46], respectively. The water absorption of the combinations of fine aggregates and FBA were 1.2% and 11.30, respectively, while bulk densities were 1650 kg/m^3^ and 950 kg/m^3^, respectively.

**Table 3:** Physical properties of FBA and fine aggregates.

Property	Unit	Fine Aggregate	FBA	Method
Water absorption	%	1.20	11.30	AS 1141.5 [44]
Clay-fine silt content	%	2.1	3.7	AS 1141.33 [47]
Specific gravity	g/cm^3^	2.50	1.45	AS 1141.5 [44]
Compacted bulk density	g/cm^3^	1.65	0.95	AS 3583.2 [46]
Uncompacted bulk density	g/cm^3^	1.41	0.89	AS 3583.2 [46]
Fineness modulus	/	4.7	4.4	/

### 2.2. Mix Proportions and Testing Setup

**Figure 5:**
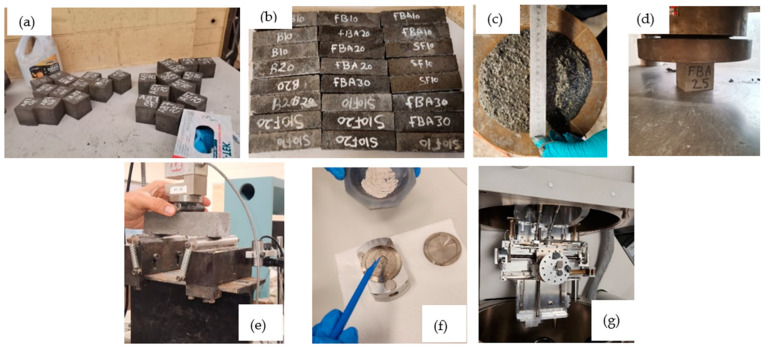
Actual photos for prepared samples and testing setup, where (**a**): mortar cubs, (**b**): mortar prisms, (**c**): flow table test, (**d**): compressive strength test setup, (**e**): flexural strength test setup, (**f**) preparing powder for XRD, and (**g**) SEM setup.

Table 4 displays the mix proportions for the proposed mortars. Firstly, four proportions of fine aggregate replacement by FBA were considered: 25%, 50%, 75%, and 100% by volume. Three essential properties of the mortar were utilized to determine the optimal percentage, including flowability, compressive strength, and water absorption. Assessing these properties was crucial for selecting the optimal percentage of FBA as a fine aggregate alternative in the mixture. These parameters were chosen because compressive strength is the primary indicator of mechanical performance, water absorption affects durability, and flowability influences rheological properties. This stage is crucial for achieving the desired balance between workability, structural integrity, and durability of the mortar, ensuring that the FBA-based mortar meets the required performance standards for construction applications. After determining the optimal level of FBA as a fine aggregate replacement, the chosen proportion was used to proceed with mortar groups using FBA as cement replacement. Three different mix formulations were developed to investigate the mortar performance with optimal FBA as fine aggregate (i.e., 25%), and 10%, 20% and 30% GFBA as cement replacements. The water-to-binder (w/b) ratio was fixed at 0.6 for all mixtures. The mortar preparation and sampling were conducted following AS/NZS 2350.12 [48]/AS 2701 [49]. 2015 specifications. Figure 5a,b shows actual photos of prepared samples. Mortar flowability (Figure 5c) was determined through the flow table test according to AS 2701 [49], while compressive strength (Figure 5d) and flexural strength (Figure 5e) were determined according to AS/NZS 2350.11 [50] and AS 2350.12 [48].

For the XRD analysis, small portions of the interior specimen tested for compressive strength were taken and ground until they passed through a 125 nm sieve, then filled into the testing disk as shown in Figure 5f. For SEM/EDS mapping, interior pieces from the tested specimen were taken and mounted on carbon tape as shown in Figure 5g. The samples were analyzed using an environmental SEM (ESEM) setup to accommodate the high-pressure chamber conditions without coating. To determine the water absorption, the mortar specimens were removed from the water tank at 28 days and kept in the lab atmosphere until the saturated-surface-dry (SSD) condition was achieved. Then, the samples were weighted, and the mass was recorded as m1. Then the mortar samples were dried in the oven at 105 °C for 24 h. After that, the specimens were cooled, the mass was recorded as m^2^, and the water absorption was calculated as:

(1) $$\mathrm{WA}(\%)\;=\frac{m_{1}-m_{2}}{m_{2}}\times 100$$

### 2.3. Environmental Impact Assessment

#### 2.3.1. Analysing Emissions

The embodied carbon dioxide (on a mass basis, i.e., CO_2_-e kg/m^3^ of mortar) for each virtual mix was calculated according to the contribution from each of its constituents, using the values given in Table 5, and described by Purnell and Black [51] and Al-Naqkeeb [52], according to the following equation:

(2) $${\mathrm{CO}}_{2}\text{-}e\;\mathrm{kg}/m^{3}\;=\sum (m_{i}\times f_{i})\;$$

where *m_i_* is the mortar material *i* in kg/m^3^ and *f_i_* is the global warming parameter equivalent to material *i* production. The *f_i_* values were obtained from Daracon group database information on materials embodied emissions sourced from Infrastructure Sustainability Materials, as shown in Table 5. The value of fi for GFBA was modified based on the treatment conditions applied to the GFBA.

#### 2.3.2. Leachability: Inductively Coupled Plasma Test

The analysis was performed in duplicate, and a standard solution calibration curve was established with a slope of 0.99 and an R-square value of 0.9999 according to AS 4439.3 [53]. The continuing calibration verification (CCV) was checked, ensuring recovery ranged from 93.64% to 100%. For quality assurance and quality control (QA/QC) purposes, a standard reference soil was analysed. Fe, Al, K, Ca, Mg and Na were analysed using Inductively Coupled Plasma Optical Emission Spectroscopy (ICP-OES), while the others were analysed using Inductively Coupled Plasma Mass Spectrometry (ICP-MS). The Limits of Reporting (LOR) for ICP-MS and ICP-OES were 0.1 ppb and 0.1 ppm, respectively.

## 3. Results and Discussion

### 3.1. Mortar Flowability

**Figure 6:**
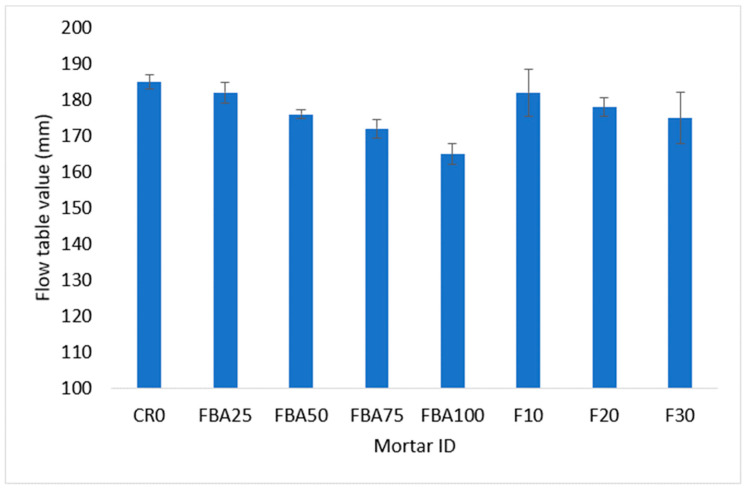
Flow diameter of designed mortars.

Figure 6 illustrates the results of a flow table test to evaluate the influence of FBA as a fine aggregate and partial cement replacement on the flowability of mortar. The results indicate that the mix with no replacement (CR0) exhibited the highest flowability; when FBA was included as a fine aggregate replacement, the mortar flowability decreased. The reduction in mortar flowability increased as the replacement level increased, with reduction percentages of 1.6%, 4.9%, 7.0% and 10.8% corresponding to the inclusion of 25%, 50%, 75%, and 100% of FBA as a fine aggregate replacement, respectively. These reductions are attributable to the high-water absorption of FBA compared to natural fine aggregate, as presented in Section 2.1. The irregular and rough shape of the FBA particles may have had an effect on increasing intermolecular friction and reducing the kinetic energy of the mortar, thus reducing its flowability [54,55]. The results are consistent with observations reported elsewhere [15,17], where the workability reduction tended to increase as the level of replacement increased. In the case of the inclusion of GFBA as a partial cement replacement, the F10 mix showed a 4.9% decrease in flow value compared to CR0, identical to the FBA25 mix. The F20 and F30 mixes experienced a 3.8% and 5.4% reduction compared to CR0 and 2.2% and 3.8% compared to the FBA25 mix, respectively. The decrease in flow diameter values compared to the control samples could have attributed to the rough and irregular surface of GFBA (Figure 1) compared to cement particles. The rough and irregular shape of the GFBA particles may have had an effect of increasing intermolecular friction, unlike the semi-smooth shape of cement particles, thus reducing the mortar flowability. Furthermore, the high fineness of GFBA particles as shown in Table 2 means they to tend to fill the inner area of the mixture particles, thus reducing the area available for particles dynamics; therefore, an excessive amount of water is needed to lubricate the mixture to give it enough flowability [56]. These findings are consistent with several studies that have reported decreased flow values in mixes containing GFBA, such as the studies by Abdulmatin et al. [23], Abbas [24], and Aydin [25]. These researchers found that the reduction in mortar flowability gradually increased with increased GFBA content.

### 3.2. Mortar Densities

**Figure 7:**
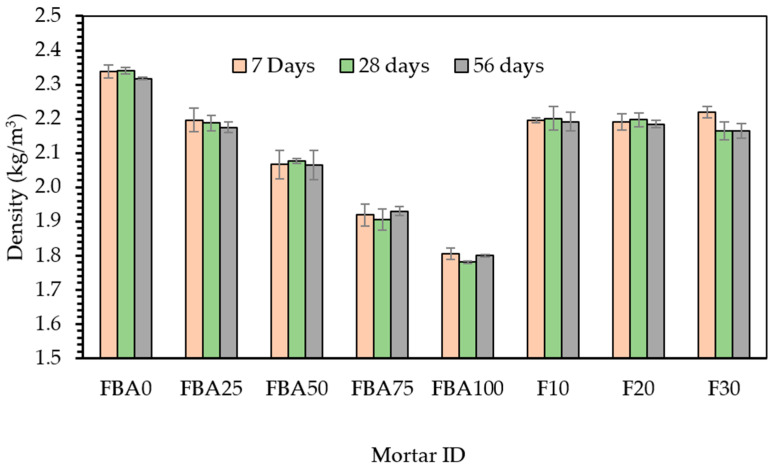
Densities of the designed mortars.

Figure 7 presents the densities of the prepared mortar samples measured at 7, 28, and 56 days, with varying percentages of FBA as fine aggregate and cement replacements. From Figure 7, the density decline is obvious in the case of using FBA as a fine aggregate. For the FBA0 mix, which had no FBA replacement, the densities were 2.34, 2.34, and 2.32 kg/m^3^ at 7, 28, and 56 days, respectively. The FBA25 mix has densities of 2.20, 2.19, and 2.17 kg/m^3^ at the respective time points, showing a similar gradual decrease. This trend was consistent across the mixes with higher FBA contents; for instance, the FBA100 mix, in which the fine aggregate was fully replaced by FBA, recorded the lowest densities across all the time points—1.81, 1.78, and 1.80 kg/m^3^. The reduction in the mortar density with the increase in FBA content might be due to the lower density of FBA compared to the fine aggregate it replaces. In the case of the inclusion of GFBA as partial cement replacement, there was no regular trend in densities. For example, F10 showed a 0.6% higher density compared to FBA25 at 28 days. This could be attributed to the filling effect, leading to a more compact matrix. The reduction extent of densities was lower than that of using FBA as a fine aggregate. However, the F20 mortar showed similar densities to those of FBA25, while F30 showed lower densities by around 1%. Compared to previous studies, Mangi et al. [57] and Oruji et al. [27] found that mortar densities decreased with increasing replacement levels of cement by GFBA. They attributed this to the lower specific gravity of GFBA compared to Portland cement. Similarly, in the case of fine aggregate replacement, several studies [58,59,60] indicated a decrease in mortar density when FBA was used as a sand substitute. This is due to the lower specific gravity of FBA, which ranges from 1.3 to 2.2 g/cm^3^, as summarized by Onaizi et al. [7], compared to approximately 2.5 to 2.65 for conventional aggregates. These findings are consistent with the results of this study, which observed a clear reduction in mortar densities with increased fine aggregate replacement due to the lower density of FBA (1.45 g/cm^3^) compared to 2.5 g/cm^3^ for the natural fine aggregate.

### 3.3. Compressive Strength

**Figure 8:**
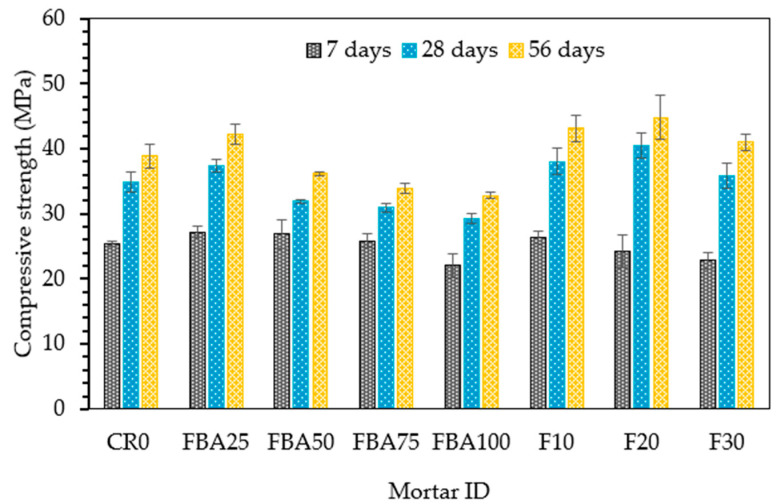
Compressive strength performance of FBA-based mortars.

Figure 8 depicts the compressive strength of the mortar samples with various replacement levels of FBA over time, specifically at 7, 28, and 56 days. The control mix (CR0), without any FBA replacement exhibited an initial compressive strength of 25.4 MPa at 7 days, which substantially increased to 34.9 MPa at 28 days and reached 38.9 MPa at 56 days. In contrast, the FBA25 mix, with a 25% replacement of fine aggregate by FBA, started with a higher initial strength of 27.2 MPa at 7 days, surpassed CR0 at 28 days by 7.2%, and achieved the highest compressive strength among all mixes at 56 days with 42.3 MPa. For the FBA50, FBA75, and FBA100 mixes, there was a slight decrease in strength at all time points compared to CR0, and the strength reduction increased with increase in fine aggregate replacement level. However, FBA50 achieved higher strength by 5.9% compared to the control sample without FBA. This could be attributed to the partial pozzolanic reaction between the fine particles of FBA and Ca(OH)_2_, resulting in the enhancement of the early strength [61]. This potentially occurs with all mixes containing fine FBA as sand; however, at high replacement levels, the negative effect of FBA porosity and weak FBA particles likely outweighed that improvement. When 10% and 20% GFBA were used as cement replacements with 25% of FBA as a fine aggregate replacement, there was more enhancement of compressive strength. The compressive strength of both mixes, F10 and F20, surpassed those of CR0 and FBA25 at 28 and 56 days. This indicates the effectiveness of co-incorporation of GFBA as partial replacements for both cement and fine aggregate. This improvement might be correlated to improvement of microstructures, which provides a more compacted and stronger bonding possibility between the binder and the aggregate. Meanwhile, the finer particles and those used as a substitute for cement reacted with the Ca(OH)_2_ produced from the initial cement hydration, providing additional hydrates [62]. The relevant studies [63,64] indicate that the improvement in the strength of cementitious mixtures is enhanced by the growth of AFt, which significantly improves the microstructures of the matrices. This was specifically observed in this study for the F10 and F20 samples (as discussed in Section 3.6). These results align with previous observations reported in the literature. For instance, a study [65] indicated that compressive strength associated with a 15% replacement level of FBA surpassed that of control specimens by approximately 17% at 28 days. Similarly, another study [66] reported that 10% GFBA increased compressive strength by around 10% at 28 days. Moreover, another study [24] noted comparable compressive strength to control specimens at 28 days for specimens made with 10% and 20% FBA. These findings suggest that finely ground coal bottom ash can improve the compressive strength of mortars by up to 20% by mass substitution compared to a control mortar without GFBA. However, these findings contradict some earlier studies [20,23,67] that reported a reduction in mortar strength regardless of the replacement ratios. It was suggested that the lower early-stage strength gain was due to the lower activity of the GFBA, which retards the cement hydration process [7,59]. However, it is important to note that these previous studies focused on the use of GFBA as a cement substitute, and there are currently no results available on the combined replacement of fine aggregate and cement.

### 3.4. Flexural Strength

**Figure 9:**
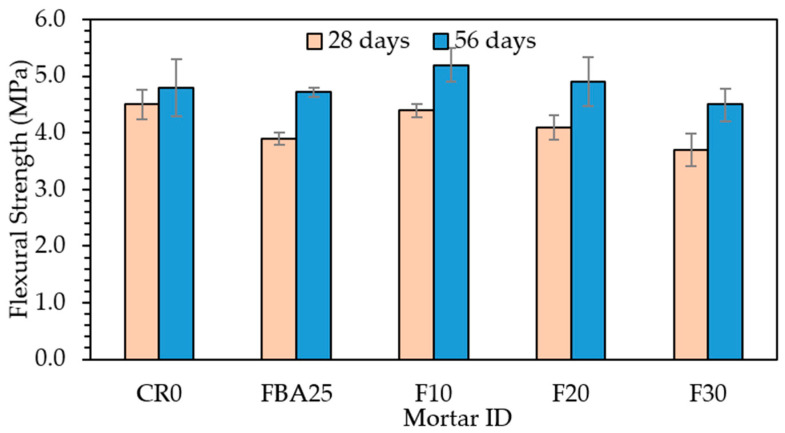
Flexural strength of the designed FBA-based mortars.

Figure 9 illustrates the flexural strength of the designed FBA-based mortar samples at 28 and 56 days. All samples containing FBA (25% of FBA as fine aggregate and 20% and 30% as cement replacements) demonstrated lower strength compared to CR0 at 28 days. For example, FBA25 demonstrated a 13.3% reduction in strength compared to CR0 at 28 days, while it showed similar flexural strength to that of CR0 at 56 days. The FBA25 sample exhibited a substantial improvement of 20.51%, with flexural strength increasing from 3.9 MPa to 4.7 MPa over the same period. At 28 days, F10 closely matched the control sample CR0, indicating comparable early strength, while F20 and F30 showed slightly lower flexural strength. After 56 days, both F10 and F20 showed significant strength increases, with F10 (5.2 MPa) being stronger than both CR0 (8.3%) and FBA25 (10.6%), proving that co-incorporating GFBA as a partial replacement for fine aggregate and cement was more beneficial. These findings are consistent with those reported in other studies [68,69,70], which demonstrated that the optimal GFBA replacements are lower than 25% and the enhancement in flexural strength usually appears after 56 days. This finding is relatively compliant with what was recorded in previous studies [24,25,34] that recorded a comparable flexural strength for control specimens at low replacement levels, with a reduction in flexural strength starting to appear at increased GFBA replacement amounts above 20%. For example, Abbas et al. [24] found that specimens with 10% FBA had comparable flexural strength to control samples, while higher FBA content resulted in reduced strength. Similarly, Kurama and Kaya [34] reported a 10.8% increase in flexural strength at 56 days with 10% FBA as a cement replacement.

### 3.5. Water Absorption

**Figure 10:**
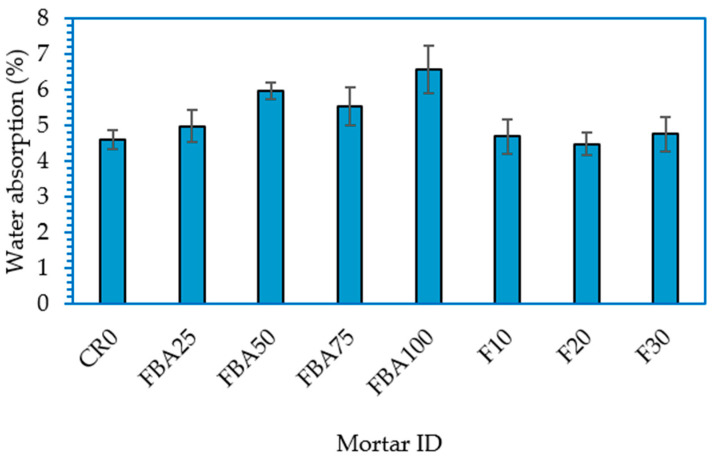
Water absorption of the designed FBA-based mortars at 28 days.

Figure 10 shows absorbed water percentages for mortar samples with FBA as partial alternatives of fine aggregate and cement at 28 days. The water absorption rate of CR0 was 4.61%. When FBA is used as fine aggregate replacement, the percentages of absorbed water increased, and the increment was boosted by increasing the replacement level. Starting with FBA25, the increment was 8.2% compared to that of CR0. This trend reversed slightly with FBA75, where water absorption decreased from 5.98 for FBA50 to 5.54% for FBA75. The higher water absorption rate was observed for the mortar sample with 100% replacement of the fine aggregate with FBA; for this sample, the water absorption was 6.58%, which was higher than that of CR0 by 42.7%. The increase in water absorption with increasing FBA content could be attributed to the intrinsic properties of FBA, particularly its porosity, which might contribute to a greater overall porosity of mortar and thus higher water absorption. However, with the co-incorporation of FBA, 25% as fine aggregate replacement and 10%, 20%, and 30% GFBA as partial cement replacements, the water absorption rates were slightly lower than the FBA25 sample. This improvement in water absorption resistance can be attributed to both the pozzolanic reaction and the filling action by the fine particles. It can be claimed that the high fineness boosted the pozzolanic reactivity of GFBA particles due to the availability of higher surface area per volume, which resulted in the C-S-H gel filling the pores, hence, reducing water absorption [39]. This finding contradicts that reported by Bheel et al. [65], who reported that the water absorption regularly decreased with an increase in the FBA content for all mixture groups. The water absorption decreased from 3.8% (for the control sample) to 1.98%, 1.75%, 1.6%, and 1.52% when cement was replaced with 10%, 20%, 30%, and 40% FBA, respectively. However, it is worth noting that the majority of studies [71,72] conducted on the use of FBA as an alternative to fine aggregate recorded an increase in water absorption due to the high porosity of FBA particles compared to natural aggregate.

**Figure 11:**
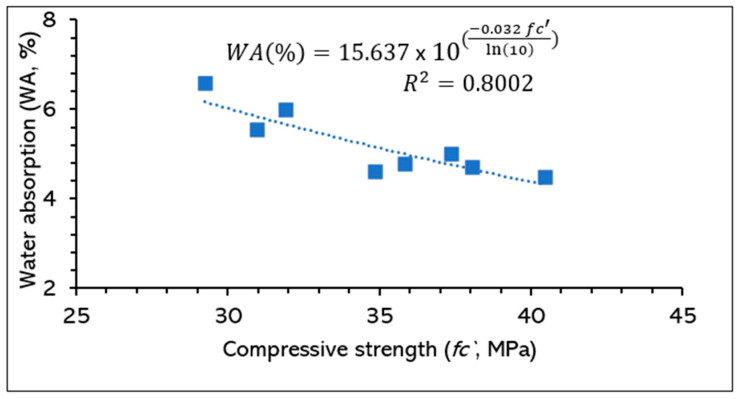
Relationship between compressive strength and water absorption of designed mortars.

The relationship between the compressive strength and water absorption of the designed mortars at 28 days is illustrated in Figure 11. The water absorption was found to be inversely proportional to compressive strength, whereby the water absorption dropped from 6.6 to 4.5% and the strength increased from 29.2 to 40.5 MPa. The exponential regression method was applied to correlate the experimental data with the R^2^ value of 0.8002, indicating a significantly good confidence in the relationships.

### 3.6. Microstructure Analysis and Discussion

**Figure 12:**
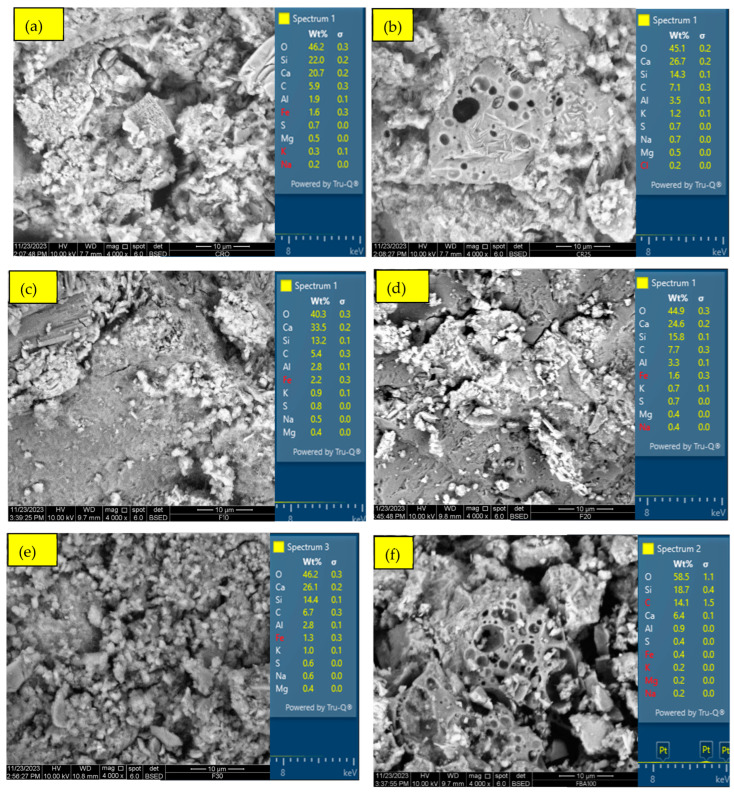
SEM imaging and sectional EDS element mapping of mortar, where (**a**) CR0; (**b**) FBA25; (**c**) F10; (**d**) F20; (**e**) F30; and (**f**) FBA100.

Figure 12 shows the SEM images of the CR0, FBA25, F10, F20, F30, and FBA100 mortars at 28 days. FBA25 shows noticeable pores in some regions within the matrix, potentially due to the presence of the more porous FBA particles. Overall, FBA25 has a more uniform microstructure than CR0. The number of these pores increased with increasing FBA content as a replacement for fine aggregate, as illustrated in Figure 12f for the FBA100 sample. When 25% FBA was used as fine aggregate replacement and 10%, 20%, and 30% ground FBA were used as partial cement alternatives, the microstructure became even more compact and uniform. This could be attributed to the finer particles of GFBA filling the pores and cracks in the mortar matrices, positively impacting the mechanical performance of F10, F20, and F30 samples.

The ability of FBA to absorb water may also play a role in reducing the available water-to-cement ratio (w/c) in the mixtures, enhancing compactness of the mortar matrixes and interfacial transition zones. Wang et al. [73] indicated that in cementitious composites, an excess amount of water beyond what is necessary for cement hydration leads to increased voids in the hardened pastes. This may explain the homogeneity of the microstructures in F10 and F20 compared to CR0. However, with the increased content of FBA as a replacement for fine aggregate, as in the FBA100 sample, or as a substitute for cement, as in the F30 sample, the negative effects of high porosity and a weaker pozzolanic reaction compared to cement gradually negated the positive effects of filling and controlling the water-to-cement ratio available for hydration and early pozzolanic reaction.

**Figure 13:**
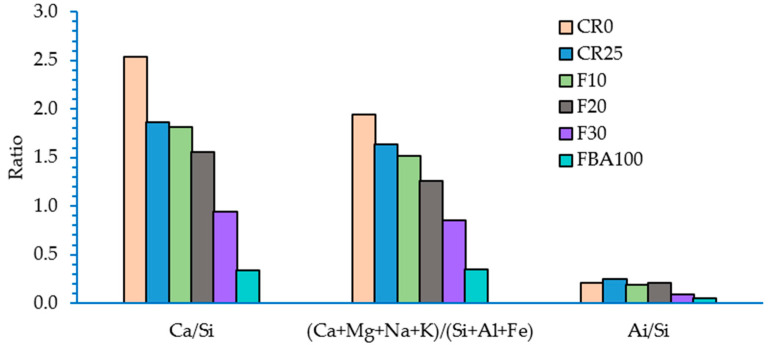
Ca/Si, Ai/Si, and (Ca + Mg + Na + K)/(Si + Al + Fe) ratios in the designed mortars.

The average elemental mapping from scanned 500-micron areas is also shown in Figure 12. The EDS mapping demonstrated that Ca decreased with increasing level of FBA either as a fine aggregate or cement alternative. In contrast, the concentrations of Si and Al increase with the increased inclusion of FBA. Figure 13 shows the ratios of Ca/Si, Ca/Al/Si/Al, and modifiers/formers network elements. The highest Ca/Si ratio was recorded for control mortar (CR0), where it was 2.54, which then dropped to 1.87 when 25% of FBA was added as a fine aggregate alternative. The drop in Ca/Si continued with the increasing inclusion of FBA, where it reached 0.94 for F30 and 0.34 for FBA100. When correlating the Ca/Si ratio to strength performance, it can be concluded that the ideal range for the Ca/Si ratio to obtain optimum strength is between 2 and 1, as shown in Table 6.

Hydration of pure Portland cement results in a C–S–H phase with a Ca/Si ratio of approximately 1.7 [74], which decreases with increasing replacement by SCMs. As SiO_2_ replacement increases, the Ca/Si ratio of C–S–H decreases significantly, ranging from 0.67 to 2.0 [75,76]. Kunther et al. [77] demonstrated that compressive strength increases with decreasing Ca/Si ratio across all samples and ages. Low Ca/Si C–S–H pastes (Ca/Si = 0.83 and 1.00) showed significant strength increases over time, whereas high Ca/Si C–S–H pastes showed only small increases. After 3 months, the compressive strength of the Ca/Si = 0.83 binder was more than double that of the highest Ca/Si ratio binder. However, the range of Ca/Si ratio in this study was between 0.83 and 1.5. Two hypotheses explain this correlation [77]:

1. Low Ca/Si C–S–H hydrates have denser microstructures, leading to lower porosities and higher compressive strengths, as lower Ca/Si ratios result in higher surface areas.
2. The density and molar volume of the C–S–H phase are correlated with the Ca/Si ratio; lower Ca/Si ratios result in lower molar volumes and higher densities, contributing to higher strengths.

Despite the consensus among researchers regarding the impact of both Ca/Si and Al/Si ratios on the mechanical properties of cementitious composites, the nature of this relationship remains debated. Some studies [78,79] suggest that the values of the modulus of elasticity and creep increase with the rise in Ca/Si ratio, which might explain the higher flexural strength of CR0 compared to other samples. Conversely, other studies indicate optimal Ca/Si ratios, deviations from which negatively affect mechanical properties. For instance, Wang et al. [80] found that the typical Ca/Si ratio, when incorporating fly ash, is around 1.4, while Garcia’s results [81] suggest a value closer to 1.8. Wang [80] also noted that the optimal Al/Si ratio is approximately 0.25, which is close to the values observed for the mortar samples with optimal performance obtained in this study: 0.24, 0.19, and 0.21 for FBA25, F10, and F20 samples, respectively. This ratio decreased to 0.09 and 0.05 for the F30 and FBA100 samples, respectively, and this decrease falls within the range defined by Wang et al. [80] as 0.5 to 0.05. Similarly, the Ca/Si ratio also declined to 0.94 and 0.34 for the F30 and FBA100 samples, respectively.

It is also important not to ignore the impact of the ratios of network modifiers to network formers due to their role in modifying pore alkalinity and participating in various complex hydrate chains. In this study, a consistent decrease in the ratio of network modifiers to network formers was observed with the increased incorporation of GFBA. This decrease might have resulted from the reduction of calcium and the increase in amorphous silica in the mixtures containing GFBA. Similar to the cases of Ca/Si and Al/Si ratios, this decrease can be beneficial to mechanical performance up to a certain point, as it involves the consumption of Ca(OH)_2_ in forming hydrate chains. However, a sharp decrease might lead to lower pore solution alkalinity, which hinders the dissolution of pozzolan particles. Previous studies indicate that increased alkali content accelerates cement hydration, enhancing strength development [82,83,84]. This acceleration occurs because alkali cations in the fresh cement mixture’s liquid phase accelerate C3A hydration by releasing Ca^2+^ cations [84,85,86]. It is also known that increasing glass network modifiers boosts the alkaline medium, enhancing dissolution of glass phase activation [87,88]. However, some studies have shown that higher alkali content can reduce concrete compressive strength [82,83,86]. This reduction is attributed to a porous microstructure and the lower strength of alkali-containing C–S–H gel in the hardened mixture [84,89,90]. This highlights the need for further research to optimize the balance between glass network modifiers and formers and the conditions that control them.

**Figure 14:**
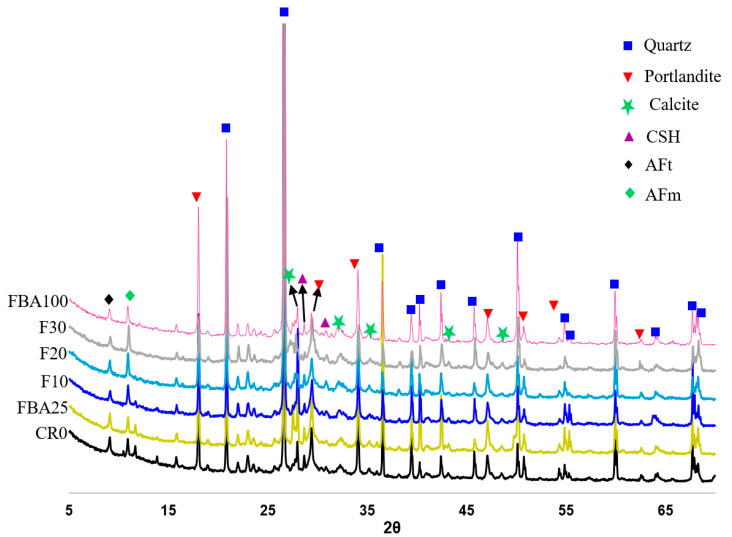
XRD patterns of the designed mortars.

Figure 14 shows the types of crystalline phases formed in the formulated mortars. As observed, six crystalline phases were detected: quartz, portlandite, calcite, calcium silicate hydrate, AFt, and monosulfate (AFm). The figure indicates an increase in the intensity of monosulfate peaks with the increase in the level of cement replacement by GFBA (Supplementary material), especially at a 30% replacement level. This increase was due to the abundance of alumina dissolved from the GFBA, which can react with ettringite in the presence of gypsum to form monosulfate. A slight decrease in AFm peaks was also observed in the outperforming samples such as F10 and FBA25, which may be attributed to its conversion to AFt. This may be due to the chemical instability of AFm, as it may react with calcium carbonate in the presence of appropriate humidity to turn into AFt [91]. Calcite was also observed, with its peaks primarily distributed between 50° and 27°. Its presence can be attributed to the relative instability of Ca(OH)_2_, which undergoes accelerated carbonation under atmospheric conditions. The quartz peaks were nearly identical in the mortar samples CR0, FBA25, F10, F20, and F30, but their intensity was more pronounced in the mortar containing a 100% replacement level of fine aggregate with FBA.

### 3.7. Environmental Impact Assessment

#### 3.7.1. Leachability

**Figure 15:**
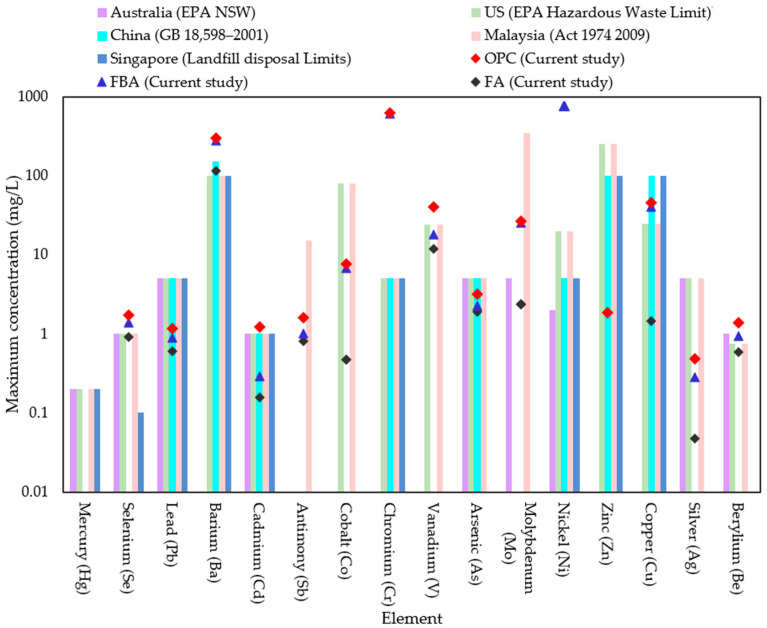
Trace element concentrations from FBA leachate and the maximum limits allowed in the relevant standards/regulations to classify solid waste as non-hazardous waste. The maximum allowable limits for Australia [95], the US [94] China [96], Malaysia [97], and Singapore [98,99] are shown in the figure.

Table 7 provides a comparative analysis of the leachability of trace elements from GFBA, OPC, and FA. The results indicate that nickel was detected in FBA but was absent in both FA and OPC, with its concentration exceeding the upper limits specified by regulations, including those of the EPA NSW, as depicted in Figure 15. Additionally, GFBA exhibited measurable concentrations of Mn, Zn, Cr, Fe, Co, Cu, Mo, Ag, Sn, and Ba, with some elements having lower concentrations than those found in FA or OPC, or both, as shown in Table 7. When comparing concentrations with the regulatory upper limits for trace elements shown in Figure 15, it becomes evident that GFBA surpasses the allowable limits for Ba, Se, Cr, and Ni. This issue is not confined to GFBA alone; certain trace elements in FA and OPC also exceeded the permissible limits established by environmental regulations. For instance, OPC exhibited concentrations of Si, Ba, Cr, and V that were higher than the allowed limits. Further research is required to understand the potential impact of these trace elements on environmental health.

However, previous studies have shown that although FBA and FA exhibit higher concentrations of some trace elements compared to relevant standards, including them in concrete bodies can immobilize their leachability. For example, Sutcu et al. [92] and Eliche-Quesada et al. [93] investigated the leaching concentrations from FBA-based fired bricks and discovered that the brick body effectively immobilized the heavy metals. Similarly, Rafieizonooz et al. [30] noted that the leaching of heavy metals from concrete containing up to 100% FBA as a sand replacement was lower than the original FBA concentration and within the US EPA’s SW-846 andard limits [94]. This indicates that the concrete body immobilized the heavy metals sufficiently, rendering them negligible. From the above, it can be concluded that the inclusion of FBA in concrete composites, similar to FA, significantly reduced the concentration of leached heavy metals compared to using FBA or FA alone.

#### 3.7.2. Equivalent Emissions

**Figure 16:**
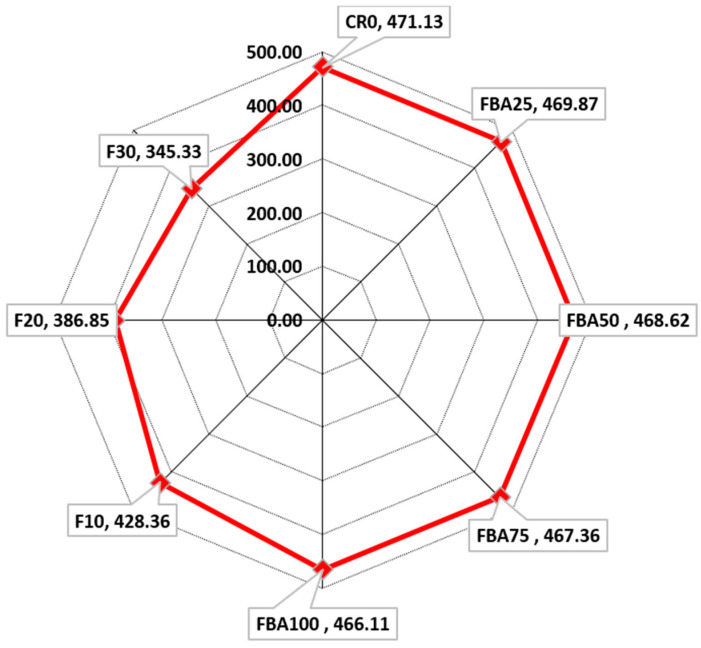
Embodied emissions (in kg/m^3^) from each mix.

Figure 16 illustrates the equivalent carbon emissions for each mortar mix. It was observed that replacing fine aggregate with FBA did not result in a significant change in the amount of embodied carbon for the FBA25, FBA50, FBA75, and FBA100 mixes compared to the control sample CR0. This is attributed to global warming parameter values of natural fine aggregate (0.00789) and FBA aggregate (0.00758). The reduction rates were 0.26%, 0.53%, 0.80%, and 1.1% when 25%, 50%, 75%, and 100% of the fine aggregate was replaced with FBA, respectively. When the replacement ratio of fine aggregate with FBA was fixed at 25% and cement was replaced with 10%, 20%, and 30% GFBA, the equivalent carbon dioxide decreased from 469.87 kg/m^3^ to 428.36, 386.85, and 345.33 kg/m^3^, respectively. This corresponds to reductions of approximately 9.1%, 17.9%, and 26.7%, respectively, compared to CR0. This is consistent with the conclusions of previous studies, which indicated that CO_2_ emissions decreased linearly with increasing FBA content as a cement replacement [100,101].

## 4. Conclusions

In this study, the impact of co-incorporating FBA as a replacement for fine aggregate and cement on mortar performance was examined, focusing on flowability, compressive strength, flexural strength, water absorption, and microstructural characteristics. The combined incorporation of 25% FBA as a fine aggregate replacement and 10% or 20% GFBA as a cement replacement significantly improved the compressive strength of the mortar after 28 and 56 days of curing, surpassing the control samples and sample with 25% of FBA as a fine aggregate replacement, though no significant variation was observed at 7 days. When the replacement level of fine aggregate exceeded 50%, notable decreases in compressive strength were observed.

The flexural strength of mixtures with 25% FBA as a fine aggregate replacement alone and 25% FBA combined with 30% GFBA as cement replacement was similar to conventional mortar at 56 days, with a slight decrease at 28 days. However, samples with 25% FBA as fine aggregate and 10% and 20% GFBA as cement replacement showed flexural strength similar to traditional mortar after 28 days and improved strength after 56 days. Water absorption rates increased significantly with the inclusion of FBA as a fine aggregate replacement, while there was no noticeable difference in water absorption with 10%, 20%, or 30% GFBA as a cement replacement. There was a notable trend of increased compressive strength with decreasing water absorption rates.

Microstructural analyses revealed denser and more compact structures in mortars with combined FBA replacement, particularly those with 25% fine aggregate and 10% and 20% GFBA as cement replacements, positively impacting their strength performance and water absorption characteristics. EDS analysis showed consistent decreases in Ca/Si and Ca/Al with increasing GFBA content. XRD analysis identified six crystalline phases in the mortars: quartz, portlandite, calcite, calcium silicate hydrate, AFt, and AFm.

In terms of environmental impact, replacing fine aggregate with FBA did not significantly change the amount of embodied carbon. However, fixing the fine aggregate replacement at 25% and replacing cement with 10%, 20%, and 30% GFBA resulted in reductions of approximately 9.1%, 17.9%, and 26.7%, respectively. GFBA exhibited lower concentrations of Mn, Zn, Cr, Fe, Co, Cu, Mo, Ag, Sn, and Ba compared to FA or OPC but exceeded the permissible limits for Ba, Se, Cr, and Ni according to regulatory standards such as the EPA NSW and US EPA for Hazardous Waste. However, based on relevant investigations conducted on concrete and bricks based on FBA, the leaching rates were lower and within permissible ranges. Further research is needed in this aspect.

## Figures and Tables

**Table 1:** Chemical compositions (wt%) of the utilized materials.

	CaO	Al_2_O_3_	SiO_2_	Fe_2_O_3_	SO_3_	Cl	K_2_O	Na_2_O	TiO_2_	MnO	LOI
OPC	69.77	4.31	17.04	3.73	3.63	0.16	0.83	-	0.24	0.17	2.23
GFBA	2.40	15.10	62.37	9.30	0.17	0.17	1.45	0.74	0.58	0.25	5.61

**Table 2:** Physical properties of OPC and GFBA.

Result Transform Type	GFBA	Ordinary Portland Cement
Specific gravity g/cm^3^	2.45	3.12
Volume weighted mean (µm)	6.17	20.37
Fineness % (passing on 45 μm)	100	82.12
Uniformity	1.28	0.75
Specific surface area (cm^2^/g)	9857	8650
Surface weighted mean	4.68	6.94
d (0.1)	1.74	3.37
d (0.5)	13.76	16.48
d (0.9)	58.92	43.44

**Table 4:** Mortar mix proportions (kg/1 m^3^).

Mixture	Sand	FBA as Sand	GFBA as Cement	Cement	w/c
CR0	1537.5	0	0	467.5	0.6
FBA25	1153.2	234.4	0	467.5	0.6
FBA50	1041.7	604.2	0	467.5	0.6
FBA75	520.8	906.3	0	467.5	0.6
FBA100	0.0	1208.3	0	467.5	0.6
F10	1153.2	234.4	46.75	420.75	0.6
F20	1153.2	234.4	93.50	374.00	0.6
F30	1153.2	234.4	140.24	327.25	0.6

Note: FBA refers to the sieved FBA to be use for fine aggregate replacement. GFBA refers to the ground FBA to be used as cement replacement.

**Table 5:** Global warming parameter values.

Material	Portland Cement	GFBA for Cement Replacement	Fine Aggregate	FBA as Fine Aggregate Rep.
kg CO_2_-e/m^3^	0.982	0.121	0.00789	0.00758

**Table 6:** Summary of Ca/Si, Ca/Al, Ai/Si, and (Ca + Mg + Na + K)/(Si + Al + Fe) and the corresponding compressive strength.

Mortar	Compressive Strength (MPa)	Ca/Si	Ca/Al	Ai/Si	(Ca + Mg + Na + K)/(Si + Al + Fe)
CR0	34.85	2.54	11.96	0.21	1.94
FBA25	37.37	1.87	7.63	0.24	1.63
FBA100	29.25	1.81	9.32	0.19	1.52
F10	38.07	1.56	7.45	0.21	1.26
F20	40.47	0.94	10.89	0.09	0.85
F30	35.86	0.34	7.11	0.05	0.35

**Table 7:** Concentrations (in mg/kg) of detected elements in FA, OPC, and GFBA.

Element	FA	GFBA	OPC
Be	0.59	0.35	0.43
V	11.9	6.2	22.4
Cr	NA	610	11.8
Mn	56	251	216
Fe	6273	44152	32292
Co	0.48	6.21	1
Ni	NA	750.86	NA
Cu	1.47	38.5	5.52
Zn	NA	NA	1.88
As	1.88	0.39	0.93
Se	0.91	0.46	0.34
Sr	43.2	34.1	68.6
Mo	2.36	23	1.08
Ag	0.05	0.24	0.2
Cd 111	0.06	0.04	0.48
Cd 114	0.1	0.09	0.46
Sn	2.89	4.71	3.21
Sb	0.81	0.2	0.58
Ba	1158	1599	205
Pb	0.6	0.3	0
Al	5428	9519	14029
K	2239	2630	3237
Ca	17962	16357	15144
Mg	978	2388	3870
Na	594	2085	3594

Note: NA indicates that the element was not detected.

## Data Availability

The raw data supporting the conclusions of this article will be made available by the authors on request.

## Supplementary Materials

The following supporting information can be downloaded at: https://www.mdpi.com/article/10.3390/ma17153834/s1.
